# Pine *(Pinus massoniana Lamb.)* Needle Extract Supplementation Improves Performance, Egg Quality, Serum Parameters, and the Gut Microbiome in Laying Hens

**DOI:** 10.3389/fnut.2022.810462

**Published:** 2022-02-10

**Authors:** Yanxin Guo, Shimeng Huang, Lihong Zhao, Jianyun Zhang, Cheng Ji, Qiugang Ma

**Affiliations:** State Key Laboratory of Animal Nutrition, College of Animal Science and Technology, China Agricultural University, Beijing, China

**Keywords:** laying hens, antioxidant, immune response, gut microbiota, pine (*Pinus massoniana Lamb*.) needle

## Abstract

The effects of Masson pine (*Pinus massoniana Lamb*.) needle extract (PNE) on gastrointestinal disorders and oxidative stress have been widely investigated using experimental models; however, the functions and mechanisms of these effects in chicken models remain unknown. We investigated the effects of Masson PNE supplementation on performance, egg quality, serum parameters, and the gut microbiome in laying hens. A total of 60 healthy 50-week-old Peking Pink laying hens with similar body conditions and egg production were randomly divided into the control (CON) (0 mg/kg PNE), PNE100 (100 mg/kg PNE), PNE200 (200 mg/kg PNE), and PNE400 (400 mg/kg PNE) groups, with fifteen replicates per treatment and one hen per replicate. Compared with the CON group, egg mass, feed conversion ratios, and yolk weight were significantly increased (*p* < 0.01) in the PNE100 group. Dietary supplementation of 100 mg/kg PNE increased the serum total protein, albumin, and glucose concentrations (*p* < 0.01) and decreased the alanine aminotransferase activity (*p* < 0.05) compared with those of the CONs. Hens in the PNE100 group had reduced serum malondialdehyde levels (*p* < 0.05) and increased catalase, superoxide dismutase, and glutathione peroxidase activities (*p* < 0.01) compared with those of the CON group. Serum proinflammatory cytokine concentrations of interleukin (IL)-1β, IL-6, and tumor necrosis factor-α were lower (*p* < 0.01) and the IL-10 level was higher (*p* < 0.01) in the PNE100 group than in the CON group. Serum immunoglobulin (Ig)A, IgG, and IgM concentrations were increased in the PNE100 group (*p* < 0.01). The relative abundance of Bacteroidetes was increased, while the relative abundances of Firmicutes and Proteobacteria were decreased in the PNE100 group. The relative abundances of *Vibrio, Shewanella*, and *Lactobacillus* were decreased, while the relative abundances of *unclassified_o_Bacteroidales, Rikenellaceae_RC9_gut_group, unclassified_f_Rikenellaceae*, and Butyricicoccaceae were increased in the PNE100 group compared with those of the CON group. PNE supplementation at 100 mg/kg improved the diversity and structure of the gut microbial composition, production performance, egg quality, and serum parameters of laying hens. The laying hens in this study had good production performance when supplemented with 100 mg/kg PNE.

## Introduction

Poultry intestinal tracts possess complex microbial communities consisting of dominant bacteria that play key roles in host performance and gut health (1). Multiple factors, such as age, production system, disease, and diet, influence the gut microbiota of laying hens (2, 3). Additionally, diet is a major factor influencing the gut microbial community (4, 5). Hence, improving poultry gut health may improve final production performance to market. Many strategies (e.g., feeding modulations, acid preparations, probiotics, and enzymes) are incorporated to protect the gut from external infections and ensure that poultry have maximum potential genetic productivity (6). Phytogenic feed additives are gaining attention for their active components and compatible biological characteristics, which have been reported to benefit animal production and health (7). Masson pine (*Pinus massoniana Lamb*.) is a highly economic tree species containing terpenoids, phenolic compounds, flavonoids, and alkaloids (8), which inhibit peroxidation (9), promote immunity (10), and confer antibacterial properties (11). One study found that adding pine needles improved quail performance and serum antioxidant systems. Broilers-fed pine powder had reduced serum total cholesterol (12) and improved meat quality. Pine needle powder increased growth performance in broilers by improving the small intestinal length and protein digestibility (13). Moreover, pine needle extracts (PNEs) have antimicrobial and anti-inflammatory activities against pathogens, stimulating growth of beneficial gut bacteria, particularly *Enterococcus* spp. and *Bifidobacterium* spp. (14, 15). However, no study has adequately demonstrated the benefits of PNEs on poultry performance, especially for laying hens.

We speculated that in late egg production, laying hens-fed PNE would show improved production performance, antioxidant abilities, and immune system and inflammatory responses compared with those of control (CON) diet-fed birds and that these effects would be associated with the benefits of PNE for the gut. Thus, this study was conducted to determine the appropriate amount of dietary PNE for laying hens according to its effects on production performance, egg quality, serum parameters, and the gut microbiota.

## Materials and Methods

The experimental protocols were approved by the institutional Animal Care and Use Committee of China Agricultural University (AW92111202-1-1, Beijing, China) and the methods were carried out in accordance with the relevant guidelines and regulations.

### Hens and Management

Laying hens were allocated to the same tier of battery cages with one hen per cage (cage size: 40 × 40 × 40 cm) and exposed to light (16 h/day) with an intensity of 16 lx. The temperature was maintained at 18–25°C throughout the experiment. Food and water were offered *ad libitum* in mash form and by nipple drinkers, respectively. All the hens remained in good health during the feeding period. No hens were culled nor any hens receive medical intervention.

### Experimental Design and Diets

A total of 60 50-week-old Peking Pink hens (Yukou Poultry Corporation Ltd., Beijing, China) were randomly allocated to one of four treatments. Previous studies fed poultry 150 mg/kg PNE; thus, we used different concentrations (100, 200, and 400 mg/kg) of PNE to comprehensively investigate the appropriate amount of dietary PNE for laying hens according to its effects on performance, egg quality, serum parameters, and the gut microbiota. Hens were fed a corn-soybean meal-based diet (Table 1) supplemented with PNE at 0 (the CON group), 100, 200, or 400 mg/kg. Each treatment consisted of 15 replicates with one bird in 15 adjacent cages per replicate. The basal diet was formulated according to the nutrient requirements for laying hens (NY/T 33-2004), published by the Chinese Ministry of Agriculture (2004). The PNE product, containing 22.11 mg/kg flavonoids and 10.50 mg/kg shikimic acid, was purchased from Shaanxi Yunqi Biotechnology Corporation Ltd., Shaanxi, China. The entire experiment lasted 9 weeks (from 50 to 58 weeks of age) consisting of a 1-week adaptation period and an 8-week experimental period.

**Table 1:** Ingredient and nutrient composition of gestation diets (%).

**Ingredients**	**Content**
Corn	56.40
Soybean meal	25.00
Corn starch	8.00
Limestone	8.30
CaHPO_4_	1.50
NaCl	0.30
DL-Methionine	0.17
Minerals premix^a^	0.30
Vitamins premix^b^	0.03
Total	100.00
**Nutrient levels**	
Metabolizable Energy (ME)/(MJ/kg)^c^	11.30
CP	15.20
Lys	0.82
Met	0.40
Met + Cys	0.64
Ca	3.56
Total P	0.54
Available P	0.35

a *1 kg of vitamin premix contained the following: VA 8,000 IU VD_3_ 3,600 IU; VE 21 IU; VK_3_ 4.2 mg; VB_1_ 3 mg; VB_2_ 10.2 mg; folic acid 0.9 mg; calcium pantothenate 15 mg; nicotinic acid 45 mg; VB_6_ 5.4 mg; VB_12_ 0.024 mg; and biotin 0.15 mg*.

b *1 kg of mineral premix contained the following: Cu 6.8 mg; Fe 66 mg; Zn 83 mg; Mn 80 mg; I 0.6 mg; and Se 0.3 mg*.

c *ME was a calculated value, while the others were measured values*.

### Performance and Egg Quality Parameters

Daily egg counts, total egg weight, and weekly feed consumption were recorded and calculated weekly as hen-day egg production, egg mass, average egg weight, average daily feed intake, and feed conversion ratio (FCR). Thirty eggs per treatment were collected for interior and exterior quality tests during the last 3 days of the end of week 58. Shell strength was measured with an egg force reader (ESTG-01, Orka Technology Ltd., Bountiful, UT, USA). Shell thickness was measured using a shell thickness tester (ESTG-01, Orka Technology Ltd.). Haugh unit, yolk color, and egg weight were measured using a multifunctional egg quality tester (EA-01, Orka Technology Ltd.). Shell color was measured with a QCR color reflectometer (QCR SPA, TSS, England, UK). The shells were weighed, the yolks were separated with a separator, and the yolk and albumin percentages were weighed and calculated.

### Blood Sample Collection and Analysis

At the end of the experiment, blood samples were collected from the wing veins of the laying hens on the same day of sampling and centrifuged at 3,000 rpm for 15 min at room temperature to separate the serum. Subsequently, serum samples were collected via pipette into 1.5-ml tubes and stored at −20°C. Serum biochemical indices, i.e., alanine aminotransferase (ALT), aspartate aminotransferase (AST), and alkaline phosphatase (ALP) activities; glucose (GLU), total protein (TP), and albumin (ALB) contents; lipid metabolism indices of total cholesterol (TC), triglycerides (TGs), low-density lipoprotein cholesterol (LDL-C) and high-density lipoprotein cholesterol (HDL-C) contents; and oxidant/antioxidant indices of malondialdehyde (MDA), total superoxide dismutase (T-SOD), glutathione peroxidase (GSH-Px), and catalase (CAT) concentrations, were determined using commercial kits (Nanjing Jiancheng Bioengineering Institute, Nanjing, Jiangsu, China). The serum cytokines, such as interleukin (IL)-1β, IL-6, IL-10, and tumor necrosis factor-α (TNF-α), were measured using the Multiskan MK3 Microplate Reader (Thermo Fisher Scientific, Wilmington, MA, USA). The serum concentrations of the immune indices, immunoglobulin (Ig)A, IgG, and IgM, were detected using the Hitachi 7600 Automatic Biochemical Analyzer.

### Gut Microbial Analysis

At the end of the experiment, six hens per treatment were selected according to body weight and euthanized via sodium pentobarbital injection to obtain their cecal contents. The cecal contents were frozen in liquid nitrogen and stored at −80°C. Cecal microbial DNA was isolated using the Hexadecyl trimethyl ammonium bromide (CTAB)/Sodium dodecyl sulfate (SDS) method and quantified using the Qubit@ 2.0 Fluorometer (Thermo Fisher Scientific). The V4 region of the 16S ribosomal RNA (16S rRNA) gene was then amplified with 515F and 806R primers with the sequences of 5′-GTGCCAGCMGCCGCGGTAA-3′ and 5′-GGACTACHVGGGTWTCTAAT-3′. DNA samples were quantified and then the V4 hypervariable region of the 16S rDNA was amplified. The final amplicon pool was evaluated using the GeneJET^TM^ Gel Extraction Kit (Thermo Fisher Scientific). Single-end reads were generated with the Ion S5 TM XL platform and filtered using the default parameters.

### Statistical Analysis

Data analyses for performance, egg quality, and serum biochemical indices were performed using the Statistical Package for the Social Sciences (SPSS) version 19.0 for Windows (SPSS Incorporation, Chicago, Illinois, USA). Data were then analyzed using one-way ANOVA and means were compared using Duncan's multiple-range test. *p* < 0.05 was considered as statistically significant. Data are expressed as the means and pooled SEM.

Single-end reads were assigned to samples based on their unique barcodes and truncated by cutting off the barcode and primer sequence. Raw reads were quality filtered under specific filtering conditions to obtain high-quality clean reads using Cutadapt (version 1.9.1). The reads were compared with the reference database using the UCHIME algorithm to detect chimera sequences, which were then removed to obtain the clean reads. Sequence analysis was performed using UPARSE software (version 7.0.1001). Sequences with ≥ 97% similarity were assigned to the same operational taxonomic units (OTUs). The subsequent clean reads were clustered as OTUs using UPARSE (version 7.0.1001) and annotated with the SILVA 16S rRNA gene database using the MOTHUR program (version 1.30.1). Alpha diversity (Shannon, Simpson, ACE, and Chao1 richness indices) was calculated based on the OTU profiles from the MOTHUR program. Bar plots and heat maps were generated with the “vegan” package in R (version 2.15.3). Principal coordinate analysis (PCoA) was performed based on the Bray–Curtis distance using Quantitative Insights Into Microbial Ecology (QIIME) (version 1.9.1). Analysis of similarities was performed to compare the similarities of the bacterial communities among groups using the “vegan” package in R (version 2.15.3). A linear discriminant analysis (LDA) effect size (LEfSe) analysis was performed to identify the differentially enriched bacterial taxa in the different bacterial communities. Finally, the correlations between key parameters and bacterial communities were assessed via the Spearman's correlation analysis using the “pheatmap” package in R (version 2.15.3). Data are expressed as mean values.

## Results

### Performance and Egg Quality Parameters

Table 2 lists the effects of dietary supplementation with PNE on the production performance of the laying hens. In the PNE100 group, the egg mass and FCR were significantly improved compared with those of the CONs (*p* < 0.05). The other performance indices differed among the groups, but not significantly (*p* > 0.05). Table 3 illustrates the egg quality parameters of hens supplemented with PNE. Dietary supplementation of 100 and 200 mg/kg PNE yielded higher yolk weights than those of the CON group (*p* < 0.05). No other egg quality parameters (i.e., shell thickness, shell strength, and Haugh units) differed among the groups at week 58 (*p* > 0.05).

**Table 2:** Effect of dietary pine needle extract (PNE) supplementation on production performance of laying hens^1^ (*n* = 15).

**Treatment**	**CON**	**PNE100**	**PNE200**	**PNE400**	**SEM**	***P*-value**
						
FI, g	101.75^a^^b^	100.30^a^	102.40^b^	102.38^b^	0.55	0.03
EP, %	82.81	90.36	88.21	86.68	2.16	0.14
EW, g	58.10	58.49	57.40	58.93	0.73	0.75
EM, g	48.08^b^	52.75^a^	50.62^a^^b^	50.87^a^^b^	0.93	0.02
FCR, g/g	2.07^a^	1.93^b^	2.00^a^^b^	1.99^a^^b^	0.02	0.03

1 *FI, average daily feed intake; EP, egg production; EW, average egg weight; EM, daily egg mass; FCR, feed-to-egg ratio*.

a,b *Within a row, means with no common superscript differ significantly (p < 0.05)*.

**Table 3:** Effect of dietary PNE supplementation on egg quality parameters of laying hens^1^.

**Treatment**	**CON**	**PNE100**	**PNE200**	**PNE400**	**SEM**	***P-*value**
Shell weight, g	10.38^a^^b^	10.21^a^	10.00^a^	10.80^b^	0.18	0.02
Yolk weight, g	25.29^a^	26.69^b^	26.69^b^	25.99^a^^b^	0.03	0.02
Shell strength, N	33.04	33.34	32.83	34.77	1.50	0.82
Shell thickness, μm	430.00^b^	412.00^a^^b^	390.00^a^	426.00^b^	0.01	0.03
Yolk color	3.30	3.30	3.35	3.40	0.14	0.96
Haugh unit	77.47^b^^c^	79.09^c^	72.98^a^	75.53^a^^b^	1.12	0.01

a,b *Within a row, means with no common superscript differ significantly (p < 0.05)*.

1 *Means were calculated using 15 replicates (two eggs per replicate) per treatment*.

### Serum Biochemical and Lipid Metabolism Indices

**Figure 1:**
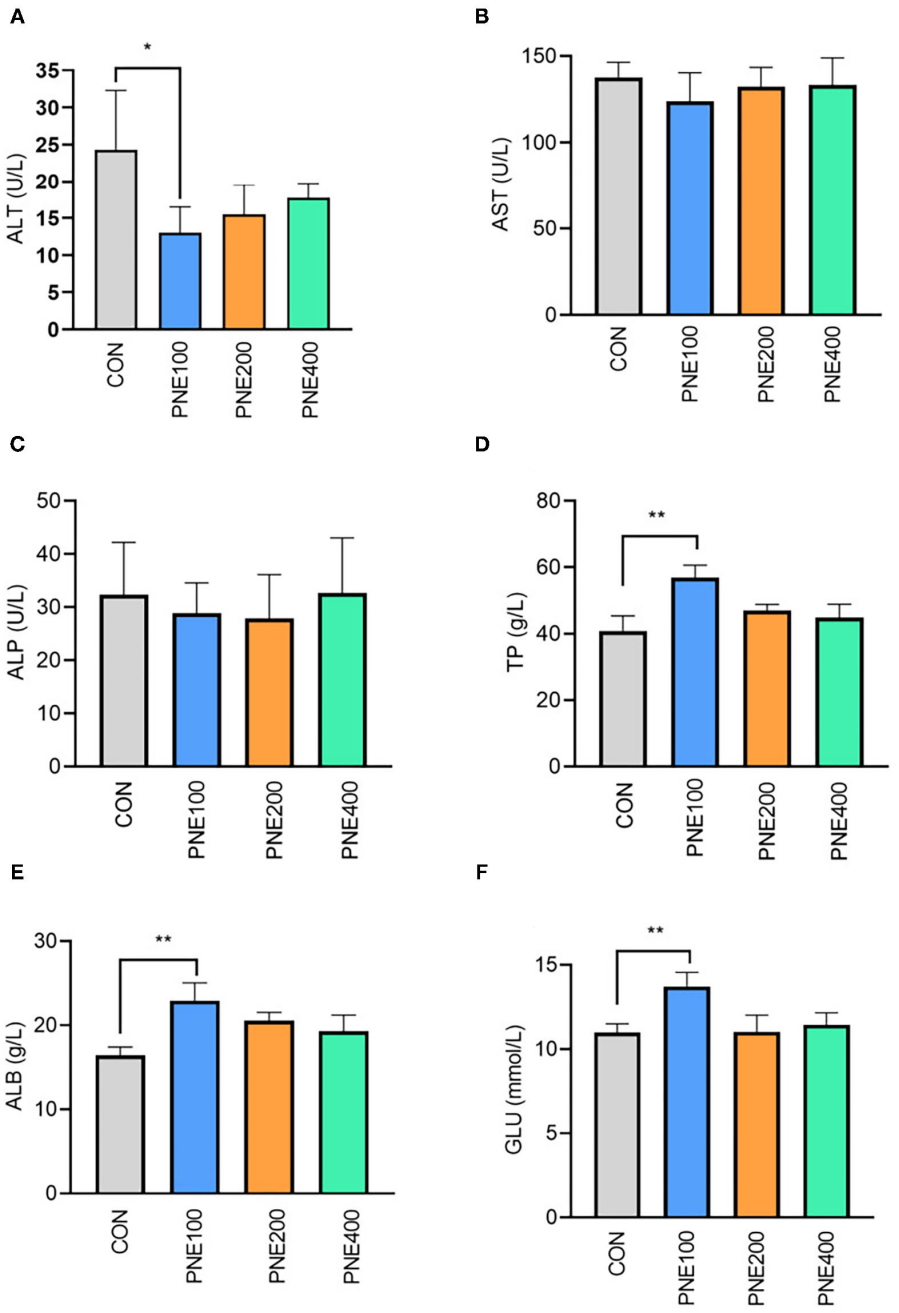
Effect of pine needle extract (PNE) supplementation on serum biochemical indices ALT **(A)**, AST **(B)**, ALP **(C)**, TP **(D)**, ALB **(E)**, and GLU **(F)** of laying hens. ALT, alanine aminotransferase; AST, aspartate aminotransferase; ALP, alkaline phosphatase; TP, total protein; ALB, albumin; GLU, glucose. Asterisks denote significant differences (*p ≤ 0.05, **p ≤ 0.01), *n* = 6 per group, data are expressed as means ± SD.

**Figure 2:**
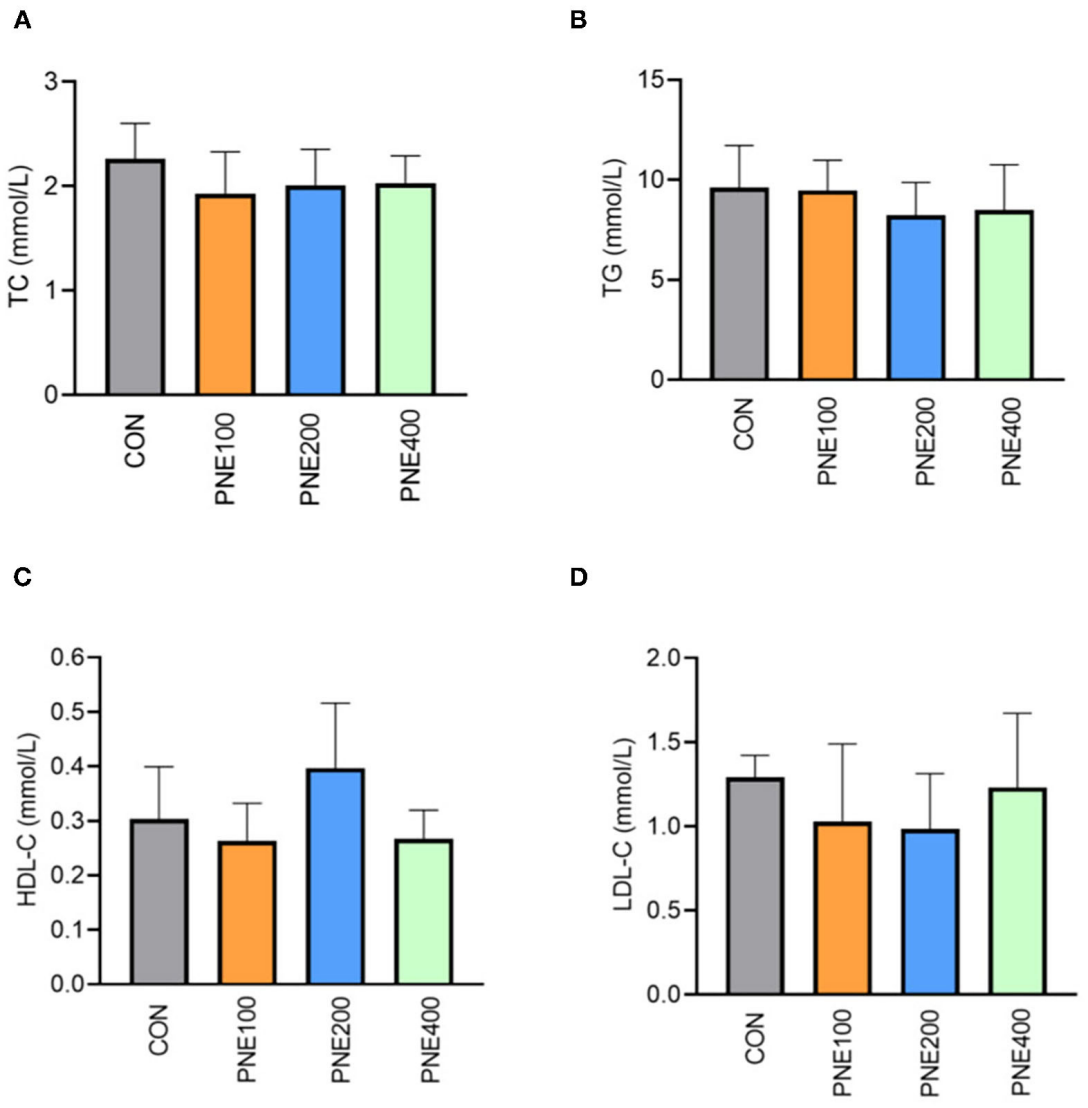
Effect of pine needle extract (PNE) supplementation on serum lipid metabolism indices TC **(A)**, TG **(B)**, HDL-C **(C)**, and LDL-C **(D)** of laying hens. TC, total cholesterol; TGs, triglycerides; HDL-C, high-density lipoprotein cholesterol; LDL-C, low-density lipoprotein cholesterol. *n* = 6 per group, data are expressed as means ± SD.

Figure 1 presents the effects of dietary PNE supplementation on serum biochemical indices. The PNE100 group had higher serum TP, ALB, and GLU concentrations than the CON group (*p* < 0.05). Compared with the CON group, ALT activity was decreased in the PNE100 group (*p* < 0.01). No other lipid metabolism indices (i.e., TC, TG, LDL-C, and HDL-C) differed (Figure 2).

### Serum Antioxidant, Cytokines, and Immune Indices

**Figure 3:**
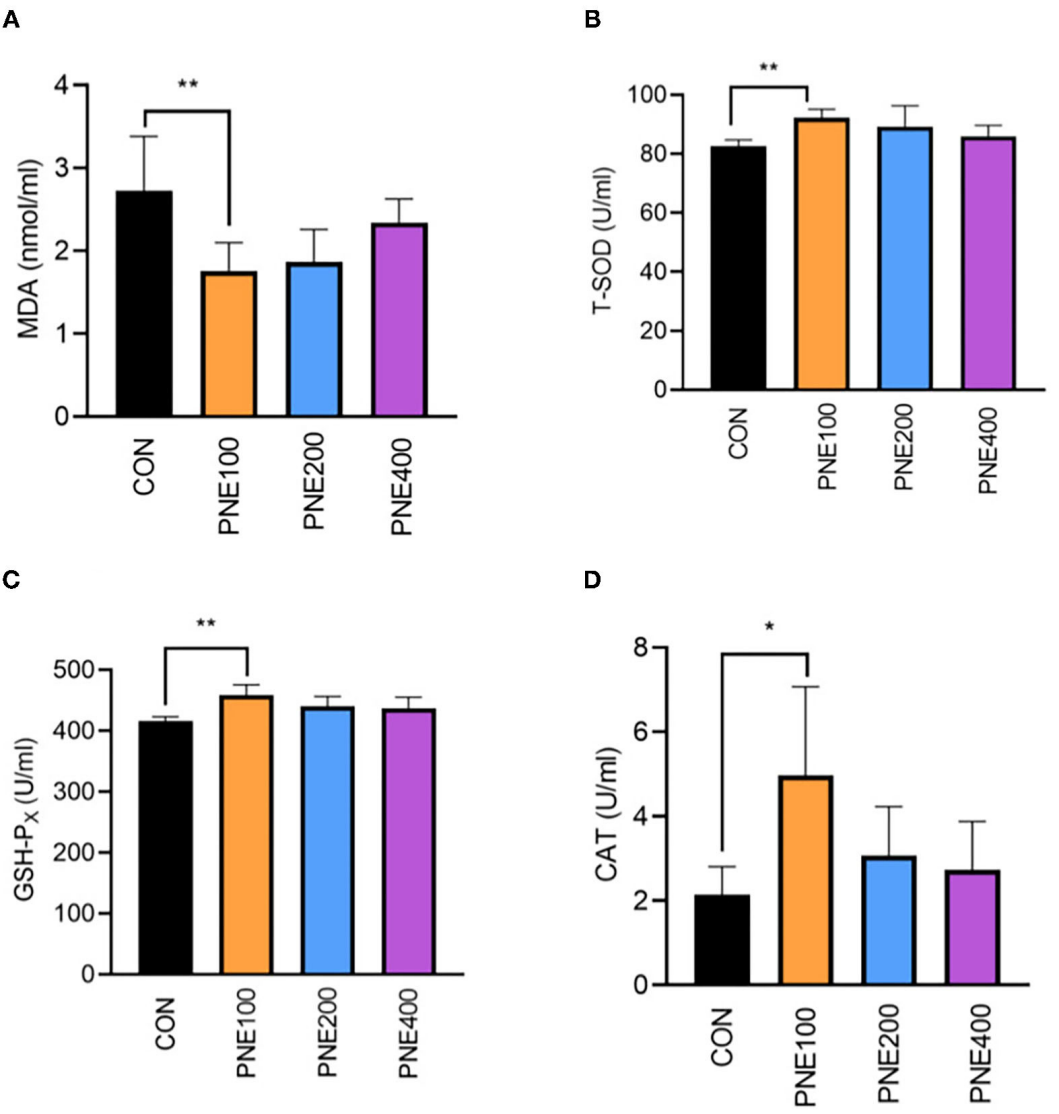
Effect of pine needle extract (PNE) supplementation on serum antioxidant parameters MDA **(A)**, T-SOD **(B)**, GSH-PX **(C)**, and CAT **(D)** of laying hens. MDA, malondialdehyde; T-SOD, total superoxide dismutase; GSH-Px, glutathione peroxidase; CAT, catalase. Asterisks denote significant differences (*p ≤ 0.05, **p ≤ 0.01), *n* = 6 per group, data are expressed as means ± SD.

**Figure 4:**
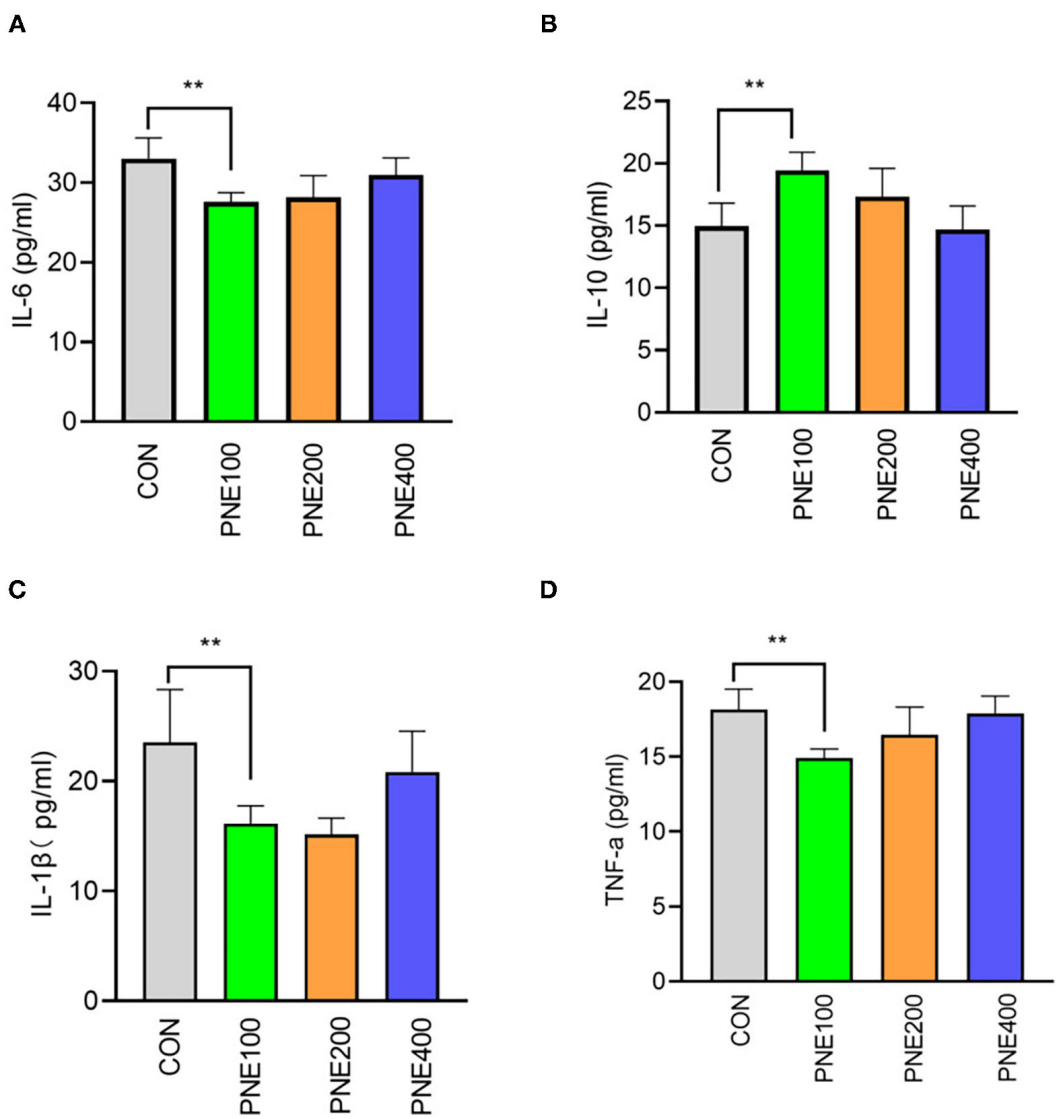
Effect of pine needle extract (PNE) supplementation on serum inflammatory cytokines IL-6 **(A)**, IL-10 **(B)**, IL-1β **(C)**, and TNF-α **(D)** of laying hens. IL-6, interleukin 6; IL-10, interleukin-10; IL-1β, interleukin-1β; TNF-α, tumor necrosis factor-α. Asterisks denote significant differences (**p ≤ 0.01), *n* = 6 per group, data are expressed as means ± SD.

**Figure 5:**
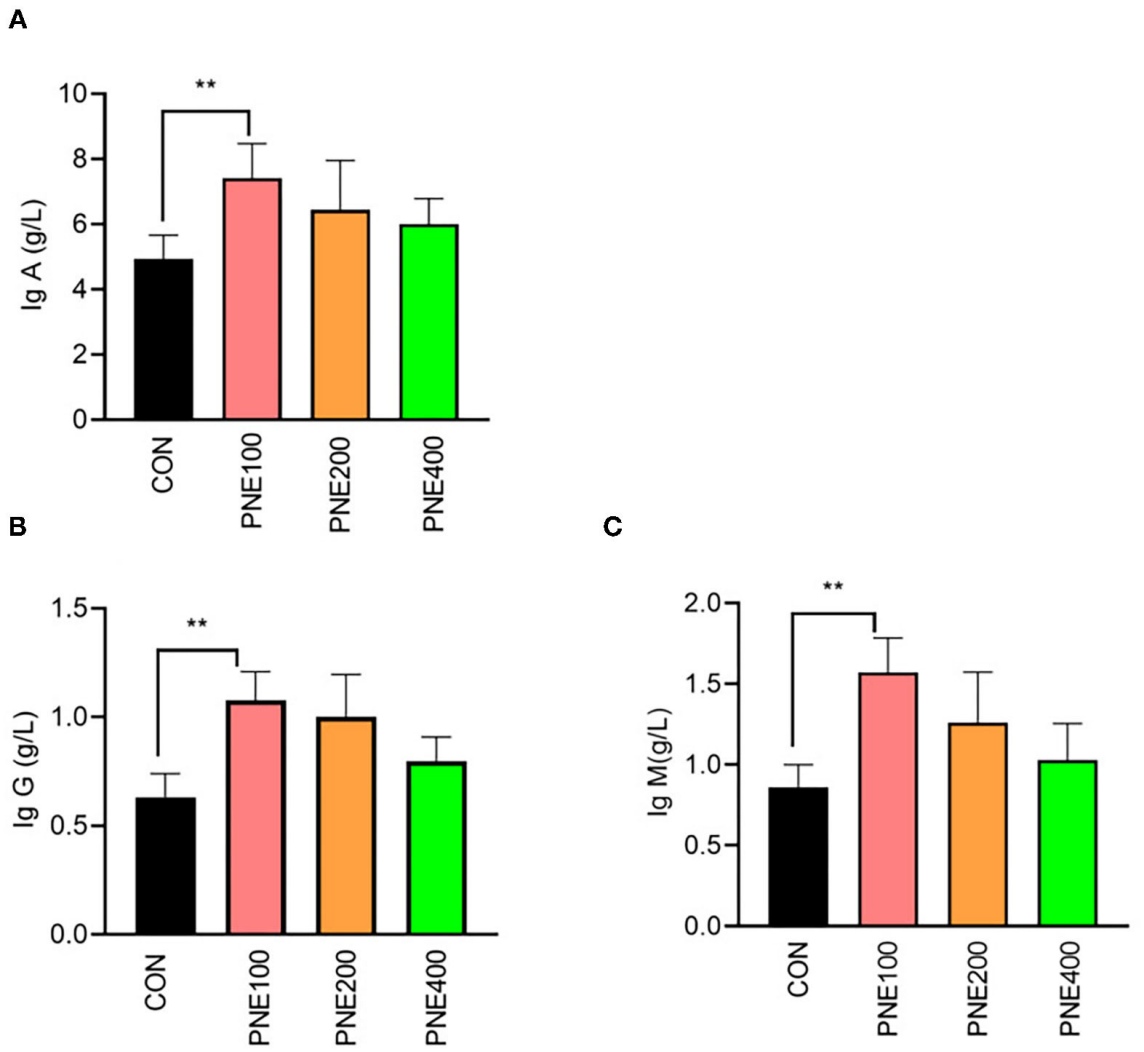
Effect of pine needle extract (PNE) supplementation on serum immune parameters IgA **(A)**, IgG **(B)**, and IgM **(C)** of laying hens. IgA, immunoglobulin A; IgG, immunoglobulin G; IgM, immunoglobulin M. Asterisks denote significant differences (**p ≤ 0.01), *n* = 6 per group, data are expressed as means ± SD.

Compared with the CON group, the MDA concentration was decreased (*p* < 0.05) and T-SOD, GSH-Px, and CAT activities were increased (*p* < 0.05) in the PNE100 group (Figure 3). Serum IL-1β, IL-6, and TNF-α concentrations were reduced (*p* < 0.01), while the IL-10 concentration was increased in the PNE100 group (*p* < 0.01; Figure 4). Dietary PNE supplementation increased the IgA, IgG, and IgM concentrations in the PNE100 group (*p* < 0.01) compared with those of the CON group (Figure 5).

### Gut Microbial Diversity and Composition

**Figure 6:**
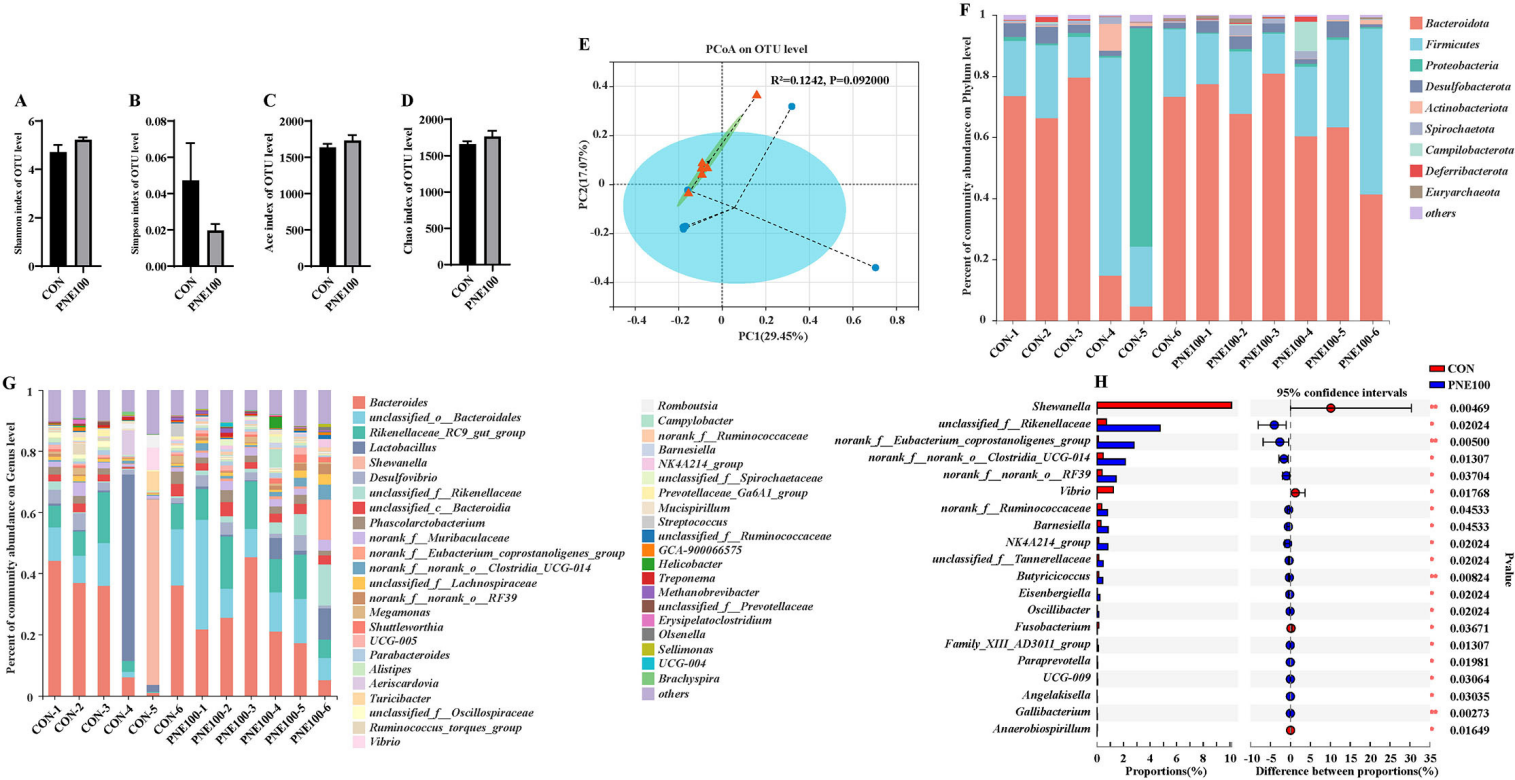
Effect of dietary PNE supplementation on gut microbial composition. **(A)** Shannon index, **(B)** Simpson richness, **(C)** ACE diversity, and **(D)** Chao1 richness. **(E)** Principal coordinate analysis (PCoA) plots were assessed via analysis of similarities among the treatments. **(F)** Relative abundances of bacterial phyla in these groups. **(G)** Relative abundance of bacterial genera in these groups. **(H)** Linear discriminant analysis effect size (LEfSe) analysis was used to identify the differential genera between the PNE100 and control (CON) groups.

Bacterial alpha diversity in the cecal microbiota was estimated using the Shannon, Simpson, ACE, and Chao1 indices of diversity and richness (Figures 6A–D). Compared with the CON group, the Shannon, ACE, and Chao1 indexes were increased and the Simpson index was decreased in the PNE100 group, but insignificantly (*p* > 0.05), suggesting that supplementation of 100 mg/kg PNE increased the overall bacterial richness of the cecal microbiota. Using PCoA based on the Bray–Curtis distance, discrimination levels were increased in the PNE100 group compared with those of the CON group (*p* < 0.10; Figure 6E). Taxonomic compositions of the microbiotas were analyzed at the phylum and genus levels. Figure 6F shows the average relative abundances (>1%) at the phylum level. Overall, the microbiotas were dominated by Bacteroidetes and Firmicutes, followed by Proteobacteria, Desulfobacterota, Actinobacteriota, Spirochaetota, Campilobacterota, Deferribacterota, and Euryarchaeota. Compared with the CON group, Proteobacteria was decreased notably in the PNE100 group, as the relative abundance of Bacteroidetes was increased and the relative abundance of Firmicutes was decreased. At the genus level (Figure 6G), the relative abundances of *Vibrio, Lactobacillus*, and *Shewanella* were decreased and the relative abundances of *unclassified_o_Bacteroidale, unclassified_c_Bacteroidia Rikenellaceae_RC9_gut_group, unclassified_f_Rikenellaceae, norank_f_Eubacterium_coprostanoligenes_group, unclassified_f_Lachnospiraceae, norank_f_norank_o_RF39, UCG-004, Helicobacter*, and *unclassified_f_Ruminococcaceae* were increased in the PNE100 group compared with those of the CON group. Compared with those of the CON group, the relative abundances of *Shewanella* and *Vibrio* were significantly decreased and *unclassified_f_Rikenellaceae, norank_f_Eubacterium_coprostanoligenes_group, norank_f_norank_o_Clostridia_UCG-014, norank_f_norank_o_RF39, norank_f_Ruminococcaceae, Barnesiella, NK4A214_group, unclassified_f_Tannerellaceae, Butyricicoccus*, and *Eisenbergiella* were increased in the PNE100 group (Figure 6H).

### Relationship Between the Differential Bacterial Community and Main Parameters

**Figure 7:**
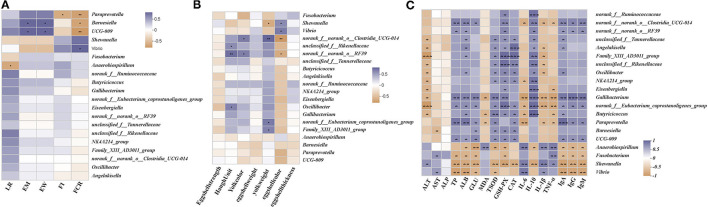
Correlation between main parameters and differential microbes. **(A)** Relationship between the differential genera and performance. **(B)** Relationship between differential genera and egg quality parameters. **(C)** Relationship between differential genera and serum parameters. Asterisks denote significant differences (*p ≤ 0.05, **p ≤ 0.01), *n* = 6 per group.

We performed the Spearman's correlation matrix to predict the correlation between the differential gut microbial communities in their genera and key parameters. Egg mass and egg weight were positively correlated with the relative abundances of *UCG-009* and *Barnesiella* and negatively correlated with the relative abundances of *Vibrio* and *Shewanella* (Figure 7A). Feed intake was positively correlated with *Vibrio, Anaerobiospirillum*, and *norank_f_Ruminococcaceae* and negatively correlated with *Paraprevotella, UCG-009*, and *Angelakisella*. The FCR was positively correlated with *Vibrio* and *Shewanella* and negatively correlated with the relative abundances of *Paraprevotella, UCG-009*, and *Barnesiella*. The Haugh units were positively correlated with *norank_f_norank_o_RF39, unclassified_f_Rikenellaceae*, and *Oscillibacter* and negatively correlated with *Shewanella* (Figure 7B). Yolk color was positively correlated with *norank_f_norank_o_RF39* and *norank_f_norank_o_Clostridia_UCG-014* and negatively correlated with the relative abundance of *Fusobacterium*. Yolk weight was positively correlated with *norank_f_norank_o_Clostridia_UCG-014, norank_f_Eubacterium_coprostanoligenes_group*, and *Family_XIII_AD3011_group* and negatively correlated with *Shewanella*. Shell color was positively correlated with *Vibrio* and *Shewanella* and negatively correlated with *norank_f_norank_o_Clostridia_UCG-014* and *norank_f_norank_o_RF39*. ALT was positively correlated with *Shewanella* and negatively correlated with *norank_f_Eubacterium_coprostanoligenes_group, Family_XIII_AD3011_group, unclassified_f_Tannerellaceae, Angelakisella, unclassified_f_Rikenellaceae, NK4A214_group, Oscillibacter, Eisenbergiella, Gallibacterium*, and *Butyricicoccus* (Figure 7C). AST was positively correlated with *Vibrio* and *Fusobacterium* and negatively correlated with *Barnesiella*. TP was positively correlated with *norank_f_norank_o_Clostridia_UCG-014, Gallibacterium, Paraprevotella, UCG-009*, and *norank_f_norank_o_RF39* and negatively correlated with *Vibrio, Shewanella*, and *Fusobacterium*. ALB was positively correlated with *norank_f_norank_o_Clostridia_UCG-014, Gallibacterium, Paraprevotella, UCG-009, norank_f_norank_o_RF39, unclassified_f_Tannerellaceae, Angelakisella*, and *Family_XIII_AD3011_group* and negatively correlated with the relative abundances of *Vibrio, Shewanella, Fusobacterium*, and *Anaerobiospirillum*. GLU was positively correlated with *Gallibacterium, norank_f_Eubacterium_coprostanoligenes_group, Paraprevotella, Barnesiella*, and *UCG-009* and negatively correlated with *Anaerobiospirillum, Shewanella, Vibrio*, and *Fusobacterium*. MDA was positively correlated with *Anaerobiospirillum* and negatively correlated with *Butyricicoccus, Paraprevotella*, and *Gallibacterium*. T-SOD was positively correlated with *norank_f_Eubacterium_coprostanoligenes_group, Paraprevotella, Barnesiella, UCG-009, unclassified_f_Tannerellaceae, Angelakisella, Family_XIII_AD3011_group, unclassified_f_Rikenellaceae, Oscillibacter*, and *NK4A214_group* and negatively correlated with *Shewanella, Anaerobiospirillum*, and *Vibrio*. GSH-Px was positively correlated with *norank_f_Eubacterium_coprostanoligenes_group, Family_XIII_AD3011_group, unclassified_f_Rikenellaceae Gallibacterium, Butyricicoccus, Barnesiella, UCG-009, Oscillibacter, NK4A214_group, Eisenbergiella, unclassified_f_Tannerellaceae, Angelakisella*, and *norank_f_Ruminococcaceae* and negatively correlated with *Shewanella, Anaerobiospirillum*, and *Fusobacterium*. CAT was positively correlated with *Family_XIII_AD3011_group, Angelakisella, unclassified_f_Rikenellaceae, Oscillibacter, NK4A214_group, norank_f_Eubacterium_coprostanoligenes_group, Butyricicoccus, Paraprevotella, Barnesiella*, and *UCG-009* and negatively correlated with *Shewanella*. IL-6 was positively correlated with *Anaerobiospirillum, Shewanella*, and *Vibrio* and negatively correlated with *Paraprevotella, Gallibacterium, norank_f_Eubacterium_coprostanoligenes_group, norank_f_norank_o_Clostridia_UCG-014*, and *Butyricicoccus*. IL-10 was positively correlated with *Eisenbergiella, Gallibacterium, norank_f_Eubacterium_coprostanoligenes_group, Butyricicoccus, NK4A214_group, Family_XIII_AD3011_group, norank_f_Ruminococcaceae norank_f_norank_o_Clostridia_UCG-014, norank_f_norank_o_RF39 unclassified_f_Tannerellaceae, Angelakisella, unclassified_f_Rikenellaceae*, and *Oscillibacter* and negatively correlated with *Shewanella* and *Anaerobiospirillum*. IL-1β was positively correlated with *Anaerobiospirillum, Shewanella*, and *Vibrio* and negatively correlated with *Gallibacterium, norank_f_Eubacterium_coprostanoligenes_group, Paraprevotella, Angelakisella, Family_XIII_AD3011_group, unclassified_f_Rikenellaceae, Oscillibacter*, and *norank_f_norank_o_Clostridia_UCG-014*. TNF-α was positively correlated with *Anaerobiospirillum, Fusobacterium*, and *Shewanella* and negatively correlated with *norank_f_Eubacterium_coprostanoligenes_group, Butyricicoccus, Paraprevotella, Gallibacterium, Eisenbergiella*, and *norank_f_norank_o_Clostridia_UCG-014*. IgA was positively correlated with *Gallibacterium, Paraprevotella, norank_f_norank_o_Clostridia_UCG-014, norank_f_norank_o_RF39 unclassified_f_Tannerellaceae, Angelakisella, UCG-009, norank_f_Eubacterium_coprostanoligenes_group*, and *Butyricicoccus* and negatively correlated with *Shewanella, Vibrio, Anaerobiospirillum*, and *Fusobacterium*. IgG was positively correlated with *Gallibacterium, Paraprevotella, norank_f_norank_o_Clostridia_UCG-014, norank_f_norank_o_RF39, UCG-009*, and *norank_f_Eubacterium_coprostanoligenes_group* and negatively correlated with *Shewanella, Vibrio, Anaerobiospirillum*, and *Fusobacterium*. IgM was positively correlated with *Gallibacterium, norank_f_norank_o_Clostridia_UCG-014, norank_f_norank_o_RF39, norank_f_Eubacterium_coprostanoligenes_group, Paraprevotella*, and *UCG-009* and negatively correlated with *Shewanella, Vibrio, Anaerobiospirillum*, and *Fusobacterium*.

## Discussion

Khan et al. reported that supplementation of 150 mg/kg PNE enhanced quail growth performance (16). Similarly, Kim et al. illustrated that broilers-fed 2.0% simple processed pine needle powder exhibited decreased overall body weight, average daily gain, average daily feed intake, and improved ileal protein digestibility compared with those of broilers-fed diets containing 0, 0.5, and 1.0% pine needle powder (13). Consistent with our results, Kothari et al. showed that adding 2.5 and 5 mg/kg fermented PNE increased egg mass and egg production (17). In this study, dietary supplementation of 100 mg/kg PNE to a corn-soybean meal-based diet improved both the FCR and egg mass of laying hens compared with those of the other groups during the trial period. Dietary supplementation of 100 or 200 mg/kg PNE also significantly improved the yolk weight. This may have been because the PNE promoted hepatic lipid metabolism by protecting hepatocytes from oxidative damage, thus enhancing the yolk weight (19), which is consistent with the results of a previous study (20). The observed improvements in egg mass and feed efficiency after PNE supplementation might have been due to the presence of essential oils, terpenoids, and polyphenols, which are reported to improve nutrient digestion, absorption, and utilization in the digestive tract (7, 18).

Several serum indicators reflect animal health status. In healthy animals, protein synthesis increases along with TP and ALB. In our experiment, serum TP and ALB were higher in the PNE100 group than in the other groups, indicating a more vigorous protein metabolism in the PNE100 group, which was consistent with the results of Kim et al. (13), who found that 1% pine needle powder strengthened the ability of broilers to utilize protein and N nutrients. Khan et al. also demonstrated that including 150 mg/kg PNE ameliorated serum TP and ALB concentrations in quails (16). ALT and AST are important biomarkers for liver function and reflect individual body conditions. In this study, birds in the PNE100 group had lower serum ALT activity, indicating that PNE may have a liver protective function. Hens in the PNE100 group had relatively higher levels of glucose and abundances of *Rikenellaceae_RC9_gut_group* than the CON group. A previous study showed that *Rikenellaceae_RC9_gut_group* was positively correlated with serum insulin levels (21), which caused higher glucose.

In this study, lipid metabolism indices (e.g., HDL, LDL-C, TG, and TC) were significantly improved in the PNE100 group compared with those of the CON, PNE200, and PNE400 groups. This was consistent with previous findings that 0.6% pine needle powder more effectively decreased mortality and improved lipid peroxidation than 0.3 and 0.9% pine needle powder (22). The improved serum lipid indices herein were also consistent with those in rats as per Lee et al. (23), likely because (24) pine needles can decrease LDL oxidation and inflammatory actions by modulating Inducible nitric oxide synthase (iNOS) and Cyclooxygenase-2 (COX-2) expressions. Additionally, 0.6% pine needle powder inhibited lipid peroxidation when supplemented in the diets of broilers (25), which was similar to our results. Khan et al. also reported that adding 150 mg/kg PNE to quail diets decreased serum LDL-C, TG, and TC concentrations and increased serum HDL-C concentrations in quails. Therefore, our results suggest that 100 mg/kg PNE was suitable for ameliorating lipid peroxidation in laying hens.

Free radicals play important roles in immunity and signal transduction, but excessive free radicals can induce lipid peroxidation in the cell membrane (26). Antioxidant enzymes such as T-SOD, GSH-Px, and CAT eliminate free radicals in the body (27); thus, antioxidant functions can be evaluated according to the antioxidant enzyme activities. Similarly, low MDA levels were shown to generate fewer free radicals in the body (28). Herein, supplementation with 100 mg/kg PNE yielded higher serum CAT, T-SOD, and GSH-Px activities and lower MDA concentrations, suggesting that 100 mg/kg PNE is suitable for elevating antioxidant functions in hens to reduce peroxide products. Chang et al. (29) found that rats fed 3 ml PNE with proanthocyanidins had higher antioxidant abilities. Khan et al. reported that supplementation of 150 mg/kg PNE ameliorated the antioxidant status in quails by enhancing the activities of serum antioxidant enzymes, including GSH-Px and T-SOD (16), which was consistent with our results. Kothari et al. demonstrated that 2.5 and 5 mg/kg fermented PNE improved the antioxidant status in broiler yolks (17), possibly because pine needles contain an abundance of active components, such as essential oils (30), proanthocyanidins (31), phenolics, and terpenoids (9), which are reported to have antioxidant abilities.

Ig G and IgM are important in anti-infection processes by engaging the phagocytic system and activating the complement system and IgA inhibits phagocytosis, chemotaxis, antibody-dependent cellular cytotoxicity, and inflammatory cytokine release (35). PNE supplementation at 100 mg/kg significantly increased the serum IgG, IgM, and IgA concentrations compared with those of other groups, which was similar to the findings of Xiao et al., who reported that pine needle polysaccharides activated macrophages and enhanced the innate immune functions of broilers (36). Wei et al. showed that 0.1% pine needle powder ameliorated the immunity of chickens (10). However, this increase was not dose dependent because birds-fed diets containing 200 and 400 mg/kg PNE showed lower immune responses than the CON group. These data indicate that 100 mg/kg PNE sufficiently induced high immune response levels in laying hens. Thus, we concluded that the improvements in egg mass and FCRs were best in the PNE100 group, likely owing to the presence of essential oils, terpenoids, and polyphenols in pine needles, which improved nutrient digestion, absorption, and utilization in the digestive tract (37).

Ig G and IgM play important roles in anti-infection through engaging the phagocytic system and activating the complement system, while IgA can inhibit phagocytosis, chemotaxis, antibody-dependent cellular cytotoxicity, and the release of inflammatory cytokines (35). In this study, PNE supplementation at 100 mg/kg significantly increased the serum IgG, IgM, and IgA concentrations compared to the other groups, which was similar to Xiao et al. reported that pine needle polysaccharide activated macrophages and enhanced the innate immune function of broilers (36). In addition, Wei et al. showed that 0.1% of pine needle powder ameliorated the immunity of chickens (10). However, this increase did not obey dose-dependent manners, when birds received the diets containing 200 and 400 mg/kg PNE showed lower immune response compared with the CON group. These data apparently indicated that 100 mg/kg PNE was appropriate to induce a high level of immune response of laying hens. From the mentioned discussion, we can conclude that the observed improvements in egg mass and FCR of hens in the PNE100 group were the best, which might be due to the presence of essential oils, terpenoids, and polyphenols in pine needles improving digestion, absorption, and utilization of nutrients in the digestive tract (37).

Next, we examined the differential cecal microbes and their functions that may have contributed to the improved performance of the hens in the PNE100 group. Alpha and beta diversity analysis showed greater cecal bacterial richness and a significantly altered microbial composition. LEfSe analysis further identified the species that differed significantly between the PNE100 group and the other treatment groups. A more diverse gut microbial community is believed to positively affect the welfare and productivity of host birds (38). *Bacteroidetes* and *Firmicutes* were the dominant bacterial phyla, which together accounted for over 80% of the total microbial community detected in this study. The gut microecosystems of PNE-fed birds were exogenously altered at the phylum level and favored *Bacteroidetes* at expense of *Firmicutes* and *Proteobacteria*, thus leading to a lower *Firmicutes*/*Bacteroidetes* ratio. Studies in mice and humans have indicated that higher *Firmicutes*/*Bacteroidetes* ratios may play important roles in energy uptake (39). Higher *Firmicutes*/*Bacteroidetes* ratios are associated with host pathology (40) and the reverse is linked to healthy gut conditions (41). Another study found that the genus *Bacteroides* in the phylum *Bacteroidetes* was positively associated with the health status of broilers (42). *Bacteroidetes* comprises many bacteria that can digest complex substrates such as xylan (43) and cellulose (44), which produce propionate (45) and succinate (46), favoring intestinal nutrient absorption in the host. *Proteobacteria* contains opportunistic pathogens, such as *Campylobacter, Escherichia*, and *Helicobacter*, which have been found to be highly abundant in the feces of low-FCR birds (47, 48). Thereby, the increased abundance of *Bacteroidetes* along with the reduced abundances of *Firmicutes* and *Proteobacteria* might contribute to the ability of laying hens to effectively absorb and use nutrients. At the genus level, adding 100 mg/kg PNE reshaped the dominant gut bacterial species. In the PNE100 group, the relative abundances of *unclassified_o_Bacteroidales, Rikenellaceae_RC9_gut_group*, unclassified_f_Rikenellaceae, and *Butyricicoccaceae* were increased and the relative abundance of *Lactobacillaceae* was decreased. *Lactobacillaceae*, which is abundant in overweight or obese individuals, is positively correlated with serum High-sensitivity C-reactive protein (hsCRP) levels and occurs in the gut microbiotas of obese persons, suggesting low-grade inflammation (47). *Rikenella*-like bacteria use mucin as a carbon and energy source and are widespread in alimentary canals of animal (48); these bacteria benefit the diversity of the intestinal flora (49). Additionally, *Rikenella* is reported to help reduce endotoxemia markers (50). *Butyricicoccaceae* are involved in energy conversion providing intestinal energy to birds. Both of these bacteria can provide energy for the body and reduce inflammation and their increased abundances indicate host intestinal health. Therefore, the improved production performance of the PNE100 group in this study may be related to increased abundances of *unclassified_o_Bacteroidales, Rikenella*-like bacteria, and *Butyricicoccaceae*, which benefit the intestinal health of the host. These increased abundances of *unclassified_o_Bacteroidales, Rikenella*-like bacteria, and *Butyricicoccaceae*, along with the decreased abundance of *Lactobacillaceae* in the guts of the PNE100 group, may have contributed to the improved performance of hens.

We used correlation analysis to predict the relationship between the main parameters and the differential gut microbiota. In the PNE100 group, egg mass was positively correlated with *Barnesiella*, which can use *fucosyllactose* as an energy source in bacterial cultures to secrete linkage-specific *fucosidase* enzymes that free lactose to form an intestinal milieu that is resilient to inflammatory diseases (53). The FCR in the PNE100 group was positively correlated with *Butyricicoccus*, which can enhance feed conversion and protect broilers (54) from potentially harmful intestinal microorganisms and necrotic enteritis. Additionally, the *Clostridial* cluster IV strain of *Butyricicoccus* is a promising probiotic candidate for patients with inflammatory bowel disease (55). The yolk weight in the PNE100 group was positively correlated with *norank_f_norank_o_Clostridia_UCG-014, norank_f_Eubacterium_coprostanoligenes_group*, and *Family_XIII_AD3011_group*. Studies have shown that *Clostridium* spp., a predominant cluster of commensal gut bacteria, exert beneficial effects on intestinal homeostasis and *norank_f_norank_o_Clostridia_UCG-014* was demonstrated to have positive effects on mouse intestinal tracts (56). Additionally, *norank_f_Eubacterium_coprostanoligenes_group* has anti-inflammatory, antioxidant, and anticancer properties (57). Similarly, Family_XIII_AD3011_group has probiotic properties and improved intestinal health in weaning piglets after inoculation (56). We observed several correlations between various serum parameters and the hen gut microbiome. TP, ALB, GLU, T-SOD, GSH-Px, CAT, IL-10, IgA, IgG, and IgM were negatively correlated with the relative abundances of *Vibrio, Shewanella, Fusobacterium*, and *Anaerobiospirillum* and positively correlated with *norank_f_norank_o_Clostridia_UCG-014, norank_f_Eubacterium_coprostanoligenes_group, Barnesiella, Paraprevotella, unclassified_f_Tannerellaceae, Angelakisella, Family_XIII_AD3011_group, unclassified_f_Rikenellaceae, Butyricicoccus, Barnesiella, Oscillibacter*, and *Eisenbergiella*. Studies have illustrated that *Shewanella* is potentially pathogenic to humans and easily infects the intestinal tract (58). *Vibrio* is pathogenic to the gastrointestinal tract, particularly in immunocompromised individuals (59, 60). *Fusobacterium* was reported to easily impede colitis remission in mice (61). *Anaerobiospirillum succiniciproducens* is a rare cause of bacteremia in humans and can cause intestinal infection (62). Interestingly, some beneficial gut microbial communities were positively correlated with these serum parameters. For example, *Clostridiaceae* Family XIII AD3011 was shown to reduce inflammation levels and alter the gut microbiotas of healthy younger men (63). *Oscillibacter* produces valerate and enhances differentiation of IL-10-producing T-regulatory cells *in vivo* (64), which improves digestive tract function (65). *Rikenellaceae* is saccharolytic and ferments glucose to acid byproducts (66). Similarly, *Parabacteroides* is a beneficial bacterium that significantly increases serum IgA and butyric acid levels in animal digestive tracts (67). *Eisenbergiella* is an anaerobic bacterium in the *Lachnospiraceae* family that produces succinate and lactate and can elevate antioxidant levels in the liver, specifically those of T-SOD, CAT, and GSH-Px compared with CONs.

Serum ALT, MDA, IL-6, IL-1β, and TNF-α are harmful indicators reflecting individual conditions. Here, these indicators were significantly lower in the PNE100 group than in the CON, PNE200, and PNE400 groups. These serum parameters were positively correlated with *Anaerobiospirillum* (62), *Vibrio* (59), *Fusobacterium* (61), and *Shewanella* (58), which are infectious pathogens. To the best of our knowledge, no study has reported the effects of pine needles on poultry intestinal flora, particularly in laying hens. However, previous reports suggest that pine ingredients such as essential oils have antibacterial properties; thus, we concluded that pine needles may have potential antibacterial effects and promote beneficial flora in the digestive tracts of hens. For example, pinosylvin (68) and essential oils (69) are components of pine that have been studied for their chemical compositions and have shown strong antimicrobial activity against common foodborne microorganisms (70). Hence, pine needles may potentially be used as natural antimicrobial agents (70). Further studies should focus on combining these effects for practical applications in laying hens. Our results showed that PNE promoted beneficial flora such as *Butyricicoccus, Barnesiella*, and *Oscillibacter* and inhibited harmful bacteria such as *Fusobacterium* and *Anaerobiospirillum*. Thus, we concluded that dietary supplementation of 100 mg/kg PNE triggered improvements in production performance and serum parameters regulated by the gut microbiome. In summary, alterations of the gut microbiome in the 100PNE group compared with those of the CON group suggested that 100 mg/kg PNE supplementation significantly modulated the cecal microbiota structure to more efficiently enhance protein utilization, protect liver function, improve lipid metabolism, boost antioxidant power, inhibit inflammatory factor expression, and increase immune responses, thus elevating production performance in laying hens.

## Conclusion

Supplementing laying hen diets with 100 mg/kg PNE induced beneficial effects on the production performance in hens. The improved performance indices (i.e., egg mass, FCR, and yolk weight) contributed to altering the gut microbial community and structure. Additionally, hens-fed diets containing 100 mg/kg PNE had better liver function with lower ALT activity, better protein metabolism, and superior oxidant/antioxidant system functioning due to increased serum CAT, T-SOD, and GSH-Px activities and decreased MDA. In summary, our results suggest that PNE supplementation at 100 mg/kg can improve the diversity and structure of the gut microbial composition, production performance, egg quality, and serum parameters in laying hens.

## Data Availability Statement

The original contributions presented in the study are included in the article/supplementary materials, further inquiries can be directed to the corresponding author/s.

## Ethics Statement

The animal study was reviewed and approved by the Institutional Animal Care and Use Committee of China Agricultural University (Beijing, China), and the methods were carried out in accordance with the relevant guidelines and regulations.

## Author Contributions

YG, SH, QM, and LZ designed the study. YG, SH, and QM conducted the experiments, drafted the manuscript, polished the manuscript, and finished the submission. YG, SH, JZ, and QM guided to analyze the experimental data, helped with revisiting, and reviewing the manuscript. All authors read and approved the final version of the manuscript.

## Funding

This study was supported by the National Science Foundation of China (Grant No. 31930105), the special fund for China Agricultural Research System program (Grant No. CARS-40-K08), and the Special Fund from Chinese Universities Scientific Fund (2018TC043).

## Conflict of Interest

The authors declare that the research was conducted in the absence of any commercial or financial relationships that could be construed as a potential conflict of interest.

## Publisher's Note

All claims expressed in this article are solely those of the authors and do not necessarily represent those of their affiliated organizations, or those of the publisher, the editors and the reviewers. Any product that may be evaluated in this article, or claim that may be made by its manufacturer, is not guaranteed or endorsed by the publisher.
